# The complexity of interpersonal physiology during rupture and repair episodes in the treatment of borderline personality disorder: a proof-of-concept multimethod single case study of verbal and non-verbal interactional dynamics

**DOI:** 10.3389/fpsyg.2024.1408183

**Published:** 2024-09-24

**Authors:** Stine S. Høgenhaug, Sune V. Steffensen, Franco Orsucci, Giovanna Zimatore, Guenter Schiepek, Mickey T. Kongerslev, Anthony Bateman, Gry Kjaersdam Telléus

**Affiliations:** ^1^Clinic North, Psychiatric Hospital, Brønderslev, Denmark; ^2^Department of Clinical Medicine, Faculty of Medicine, Aalborg University, Aalborg, Denmark; ^3^Danish Institute for Advanced Study, Odense, Denmark; ^4^Center for Ecolinguistics, South China Agricultural University, Guangzhou, China; ^5^College of International Studies, Southwest University, Chongqing, China; ^6^Department for Culture and Language, University of Southern Denmark, Odense, Denmark; ^7^Norfolk and Suffolk NHS Foundation Trust, Research & Development Hub, Norwich, United Kingdom; ^8^CEMHS – Centre for Excellence in Mental Health Sciences, University of Amsterdam, Amsterdam, Netherlands; ^9^Department of Theoretical and Applied Sciences, eCampus University, Novedrate, Italy; ^10^CNR Institute for Microelectronics and Microsystems (IMM), Bologna, Italy; ^11^Institute of Synergetics and Psychotherapy Research, Paracelsus Medical University Salzburg, Salzburg, Austria; ^12^Department of Psychology, University of Southern Denmark, Odense, Denmark; ^13^Mental Health Services West, Region Zealand, Slagelse, Denmark; ^14^Research Department of Clinical, Educational and Health Psychology, University College London, London, United Kingdom; ^15^Psychiatry, Aalborg University Hospital, Aalborg, Denmark; ^16^Psychology, Department of Communication and Psychology, Aalborg University, Aalborg, Denmark

**Keywords:** interpersonal physiology, process research, recurrence quantification analysis, determinism, complex dynamics, alliance, rupture, repair processes

## Abstract

**Introduction:**

The aim of this proof-of-concept multimethod exploratory single case study is to increase knowledge of the underlying mechanisms of alliance ruptures and repairs in Borderline Personality Disorder treatment across and within the psychotherapeutic treatment process.

**Method:**

The multimethod includes outcome assessment of patient self-reporting questionnaires (the Affect Integration Inventory, the Hopkins Symptom Checklist), observation-based ratings of sessions with the Rupture Resolution Rating System, quantitative analysis of heart rate variability using recurrence quantification analysis, and a qualitative multimodal interaction analysis of within-session dynamics.

**Result:**

Results reveal how patterns of heart rate synchrony between patient and therapist reflect periodical patterns of emotional interaction corresponding to key therapeutic alliance processes throughout the treatment process. Particularly, heart rate synchronization and desynchronization correspond with increasing rupture resolution ratings and positive outcome measures in the last part of the therapy process, indicating increased productivity, affectivity, and positive change. The qualitative microanalysis highlights context sensitivity to alliance management within sessions. Physiological arousal is found to underlie important alliance processes, including emotion regulation, relatedness, security, empathic responding, sense-making, and validation in correspondence with different therapist verbal and non-verbal markerbs.

**Discussion:**

Clinical implications and study limitations are discussed. Recommendations are made for future directions in relation to applying multimethod approaches when studying rupture and repair processes in psychotherapy.

## 1 Introduction

Pervasive patterns of emotional instability and dysregulation, identity disturbance, lack of behavioral control, impulsivity, and interpersonal dysfunction characterize borderline personality disorder (BPD) (Gunderson, 2011). BPD is associated with insecure or disorganized attachment, impaired mentalization (the ability to understand the mental states of self and others), and reduced epistemic trust (the ability to take in new social knowledge from others) (Fonagy and Luyten, 2009). Patients with BPD have a compromised capacity to engage in *we-experiences*—a process of thinking together, where the interacting partners have an experience of being part of a set of thoughts and feelings beyond their own (Bateman et al., 2021). They have a decreased ability to engage in joint attention, joint intentionality, and coordination of perspectives in the therapeutic interaction (Fonagy and Allison, 2014). Thus, the pathology of BPD may challenge the development and maintenance of the therapeutic alliance and progress in treatment (McMain et al., 2015).

Patients with high levels of personality disorder traits are more likely to have an unstable relationship with their therapists compared to patients with low levels of personality disorder traits (Fonagy et al., 2023). The quality of the alliance is a specific focus of attention in all effective treatments for BPD (Storebø et al., 2020). The alliance is found to be a relatively robust predictor of outcome, and alliance improvement is even seen as progress in itself (Bo et al., 2022). Nevertheless, information on the underlying mechanisms involved in alliance development is still lacking and highly needed to qualify treatment protocols, supervision, and training.

Based on the transtheoretical concept of Bordin (1979), the alliance is understood as collaboration on common goals and tasks and the development of an emotional bond between patient and therapist. The alliance is a dynamic process where the patient and therapist are mutually responsible for the therapeutic process (Safran et al., 2011). Special attention has been given to phases of ruptures and repairs. Alliance ruptures occur when there is a lack of collaboration on common goals or tasks, and it manifests as tensions or breakdowns in the therapeutic interaction, influencing the relational bond (Eubanks et al., 2018). Ruptures are organized into two subtypes: confrontation and withdrawal. Confrontation ruptures include direct complaints or criticisms, where the patient or therapist moves against the other. Withdrawal ruptures are sequences of moving away or disconnecting from the other (Eubanks-Carter et al., 2014). Repair is described as exploring and working through ruptures, resulting in the re-establishment of the relational bond and collaboration on goals and tasks of treatment. Proper management of ruptures is associated with better alliances and outcomes, while the failure to address and repair ruptures is associated with premature dropout, lack of progress, and deterioration (Eubanks et al., 2018).

Emerging evidence shows a higher intensity and greater number of BPD treatment ruptures than those without BPD (Gersh et al., 2018; Schenk et al., 2021). The developmental process of ruptures in BPD treatment has been found to follow an inverted U-shape trajectory, where most ruptures occur in the middle of a treatment process (Schenk et al., 2019). However, individual differences have also been identified, showing rupture occurrence intensively in single sessions or within phases of successive sessions. According to rupture types, one study found confrontation ruptures to occur more frequently than withdrawal ruptures in the initial phase of therapy in patients recovered from BPD compared to patients unrecovered from BPD (Boritz et al., 2018), while another study found withdrawal ruptures to be more frequent in the initial phase of BPD therapy and confrontation ruptures occur more often in the last phase of therapy (Gersh et al., 2017). Schenk et al. (2019) reported withdrawal ruptures to occur more frequently than confrontation ruptures, while confrontation ruptures were found to have a greater impact on the therapeutic alliance than withdrawal ruptures in BPD therapy. Gersh et al. (2017) argued that ruptures in the initial phase of therapy are more likely to be problematic, while ruptures occurring in the last phase of therapy could be opportunities for therapeutic change.

Examinations of the developmental process and the mechanisms involved in rupture and repair sequences are complex, and no golden standard exists for operationalizing them. Consequently, multiple research methods have been applied, including indirect self-rated questionnaires, direct self-rated questionnaires, and observer-based assessments of transcriptions and videos from therapy sessions (Schenk et al., 2021). The methods have generally relied on subjective and explicit observations made by a therapist, patient, or observer, tending to favor explicit collaboration while overlooking the significant role of implicit and unconscious factors (Kleinbub et al., 2020). This underscores the need for further development in methodologies, especially concerning interactional processes that are challenging to identify from a subjective perspective (Orsucci et al., 2006). Thus, this single case study aimed to illustrate the potential of applying a multimethod approach integrating both explicit verbal and observable processes and implicit non-verbal processes over the complete treatment course and within single sessions to elevate our understanding of change mechanisms in a more detailed and multifaceted way (Gelo et al., 2008; Trasmundi et al., 2023).

While nomothetic approaches examining alliance processes have their strength in pointing out global correlates between therapists’ and patients’ characteristics, they cannot fully describe the fluctuating nature of within-session dynamics of the therapeutic process. For instance, measures of tendencies for a group cannot be assumed to reflect the single individual within the group and may tend to obscure clinically relevant individual differences (Barlow and Nock, 2009). Aggregating data across individuals or sessions may tend to oversee singular characteristics that may be determinant for a specific context (Kramer et al., 2023; Zilcha-Mano and Ramseyer, 2020). According to this rationale, which change processes are most central, and how change processes unfold over time cannot be presumed to be homogeneous across individuals (Hofmann et al., 2020; Steffensen, 2016). Hence, idiographic multi-modal examinations of micro-processes between the therapist and patient within the therapeutic interaction are vital to help identifying and addressing weaknesses in the alliance to increase our understanding of the mutual contribution and responsibility of the patient and therapist according to alliance formation and interactional characteristics important for working through challenging moments of interaction in ways that may support developmental growth (Safran et al., 2001; Samstag et al., 1998). It is worth noticing, however, that idiographic studies can be aggregated and included in the examination of nomothetic research question and theory testing (Hayes et al., 2019).

The gain of integrating a multimethod embodied perspective, including both verbal and non-verbal perspectives throughout treatment and within single sessions, is the possibility of detailed examinations of the mutual transformation and emotion regulation processes in both the patient and therapist when navigating the alliance. Such investigation may help uncover and make implicit processes more explicit, which might, in future work, help identify therapeutic strategies to manage crisis, which could be integrated into our clinical models (Koole and Tschacher, 2016). Additionally, combining methods might reveal valuable tools for detecting important change processes during therapeutic interaction (Gelo et al., 2015). Potentially, this could lead to new automated methods identifying crucial moment-to-moment fluctuation in the clinical process for common investigation in supervision and training of psychologists to guide the identification and addressing of rupture and repair processes with their patients (Kleinbub, 2017). Such knowledge is highly needed, as current research shows how therapists, no matter their clinical level of expertise, have a hard time identifying and addressing ruptures, leaving many ruptures unspoken and unhandled, which naturally challenges the security and trust in the therapeutic collaboration (Hill et al., 2001; Safran et al., 2011). Moreover, adult patients with BPD show less commitment repairing the alliance when exposed to rejection compared to a group of healthy controls (Michael et al., 2021).

A growing interest has emerged in studying the more implicit aspects of the alliance, including the examination of physiological synchronization between the patient and therapist (Orsucci et al., 2016; Wiltshire et al., 2020). However, studying physiological synchronization is complex, and the terminology surrounding it is diverse, including concepts like concordance (Marci et al., 2007), embodied synchrony (Karvonen et al., 2016), interpersonal physiology (Palumbo et al., 2017), and physiological synchronization (Tal et al., 2023). In this study, the term *interpersonal physiology* (IP) was applied and defined as a shared temporal organization of physiological signals of two or more interacting people (Palumbo et al., 2017). IP has been grounded on research in developmental psychobiology in processes of attachment and separation in animal models of mother-infant interaction, which led to the discovery of “hidden regulators” in the mother-infant interaction and provided a developmental embodiment mechanism that could mediate the long-term effects of early experience (Hofer, 1994, 2006).

Understanding the concept of IP and its clinical implications might become more comprehensive when viewed through a developmental lens. From birth, infants and their caregivers form interacting patterns that include movement synchronization, facial expressions, and physiology (Beebe et al., 2003). The caregiver has a predisposition to respond contingently to the infant’s expressive displays, adapting to the infant’s rhythms and cycles of behavior, setting the scene for the development of IP (Timmons et al., 2015). Through “marked” mirroring interactions, the caregiver signals their referential emotion displays to the infant to teach the infant about its subjective experiences (Fonagy and Target, 2007). These markings are presented in different behavioral communicative channels (modalities) and inform the infant that the concurrent mirroring of affect is generalizable, relevant, and safe to listen to, which trigger an openness to learning (epistemic trust) (Fonagy and Allison, 2014). As a part of the interactive “dance” between caregiver and infant during these marked mirroring interactions, IP has been shown to play a crucial role in emotion regulation, the feeling of safety, sense-making, empathy, learning, joint attention, and trust (Feldman, 2007). IP helps children internalize emotional security also when the caregiver is absent, enabling the child to self-regulate emotional distress in different contexts (Beebe et al., 2003). It has been hypothesized that IP patterns developed in early attachment relationships might transfer to close relationships in adulthood (Timmons et al., 2015). This is interesting when studying patients with BPD, who often have traumatic narrations with lack of “good enough” marked mirroring interactions, a high degree of dyadic mismatching, and an absence of recalled reparations which might lead to increased epistemic hypervigilance (Bateman and Fonagy, 2003). As shown in childhood developmental research, IP may play an important role during dyadic mismatching in therapeutic interactions with BPD patients and may function as a facilitator of repairing tension by supporting the process of generating a sense of being and thinking together. In that way, supporting a “we-mode,” which may serve as a trigger for epistemic trust, encouraging the patients to “turn off” vigilance and take in new knowledge as something trustworthy and relevant (Fonagy and Allison, 2014). This aligns with studies suggesting that people synchronize more to people they trust and want to learn from (Beebe and Lachmann, 2020). IP has been associated with various psychosocial constructs such as empathy (Marci et al., 2007), stress contagion (Waters et al., 2014), attachment security (Diamond and Fagundes, 2010), therapeutic alliance (Karvonen et al., 2016), and therapeutic change (Tourunen et al., 2020) in adult psychotherapy. Overall, these findings indicate that IP is associated with key clinical variables that represent a measure of the interactional process. The findings underline the potential value of studying IP as a possible provider of insight into the underlying mechanisms of alliance formation, negotiation, and management. Studying the association between IP and rupture and repair episodes might allow the bridging of concepts like synchronization, emotion regulation, trust, safety, and learning during the therapeutic interaction. A recent review examining the association between rupture repair processes and interpersonal coordination in psychotherapy found synchronization between patients and therapists in different behavioral modalities of vocalization, facial expressions, movement, physiology, and hormones to be associated with rupture and repair processes (Høgenhaug et al., 2024). Moreover, interpersonal coordination was found to underline important alliance processes during rupture repair episodes, including mutual emotion regulation, sense-making, trust, and safety. High heterogeneity was identified per how interpersonal coordination was associated with rupture repair sequences, calling for further examination (Høgenhaug et al., 2024).

This study examined IP by analyzing the heart rate output (HR) as a proxy of emotional interaction, communication, and regulation. As a core challenge in the pathology of BPD is emotional dysregulation, studying HR dynamics may help reveal important implicit emotion regulation processes that are hard to see in the observable and explicit process of interaction between patient and therapist (Glenn and Klonsky, 2009). HR is related to both sympathetic activity (SA), including fight or flight responses, the orientation response, arousal activation, and attention, as well as parasympathetic (PS) activity involving resting, feeding, and sexual arousal, as well as emotional, attentional, and cognitive processes (Smith et al., 2017). The sympathetic and PS nervous systems work harmoniously to maintain the autonomic nervous system’s (ANS) control over HR. Acceleration of the heartbeat is caused by increased SA or decreased PS activity (Rajendra Acharya et al., 2006). Conversely, cardiac deceleration is brought on by low SA or increased PS activity. The respiratory control centers, which modify the vagal outflow in the brainstem, maintain an additional fine HR regulation (Giuliani et al., 1998). HR and heart rate variability (HRV), which the ANS firmly controls, can give important information about the dynamics and regulatory mechanisms of the ANS. Exploring HR patterns provides an intuitive avenue to gain insights into the underlying mechanisms associated with rupture and repair dynamics. It can be studied with high temporal resolution that allows the examination of the clinical process on a moment-to-moment basis (Zimatore et al., 2021; Zimatore et al., 2022; Zimatore et al., 2023). It enables objective measures of processes not consciously controlled or visually assessed in the therapeutic process or in observer-based ratings, making it particularly interesting to connect with the study of more observable and verbal interaction processes (Koole and Tschacher, 2016).

This study aimed to deepen our understanding of the underlying mechanisms of change in the therapeutic alliance. This was done using a proof-of-concept multimethod exploratory single case study design to examine how IP might relate to the alliance rupture and repair processes throughout treatment and on a micro-level within single sessions. The following research questions were examined to answer the overall aim: Research question 1 (RQ1): How does rupture and repair processes develop over the course of treatment. Research question 2 (RQ2): How does IP develop throughout treatment. Research question 3 (RQ3): How do IP processes relate to rupture and repair development for treatment, and research question 4 (RQ4): How does IP reflect the multimodal therapeutic interaction within sessions. Although the study is exploratory, the goal is to illustrate the importance and potential of process research to create and develop multifaceted understandings of change mechanisms in BPD and their treatment.

## 2 Methods

### 2.1 The sample

The data were drawn from a larger data pool from *The Ecology of Psychotherapy: Integrating Cognition, Language, and Emotion* (EPICLE) study (University of Southern Denmark). The data were collected at an outpatient clinic for patients with anxiety disorders and personality disorders in the North Denmark Region. In total, 30 patients (10 patients suffering from anxiety disorders and 20 patients suffering from personality disorders) were included in the EPICLE study and completed their treatment between March 2017 and January 2020.

EPICLE was reported to the Danish National Committee on Health Research Ethics and the Data Inspectorate, Journal No. 2015-57-0008. Upon inquiry to the North Denmark Region Committee on Health Research Ethics, it was relayed that research ethics approval was not needed due to the nature of the project.

#### 2.1.1 The case

The case selected for this study is an example of a successful treatment. The selection was based on the following inclusion criteria: (1) a successful outcome defined by clinical cut-off scores in pre-post treatment measured by outcome measures of the Hopkins Symptom Checklist (SCL-92) (Lambert and Ogles, 2009) and Affect Integration Inventory (AII) (Solbakken et al., 2017); and (2) data availability concerning video material (two-thirds of the video recordings), and physiological data (two-thirds of the signals; an overview is provided in the Supplementary material). One patient-therapist dyad met the criteria and was included for further analysis.

The patient completed the SCL-92 and the AII five times during therapy and at follow-up 6 months after the end of treatment. The SCL-92 is a well-established, validated questionnaire used as an outcome measure for psychological and affective distress reported by clients. It consists of 92 questions and provides a global total score called the Global Severity Index (GSI). The items on the scale are rated on a 5-point Likert scale ranging from 0 (*not at all*) to 4 (*extremely*). Lower scores indicate lower levels of symptomatology. The GSI is calculated as an average score across 90 items. Studies of Danish normative samples have found a raw score cut-off for the GSI at 1.08 for females and 0.87 for males (Olsen et al., 2006). The time frame for answering is the past week.

The AII is a validated self-report instrument measuring the functional and fluent integration of affect, cognition, and behavior (Solbakken et al., 2017). The AII consists of 112 statements about perceived awareness. The items are rated on a 10-point Likert scale ranging from *does not fit at all* (0) to *fits perfectly* (9). AII scores are divided into three sub-levels, including an overall mean score across all items (Global AI), a mean score for experience of affect (82 items), and a mean score for expression of affect (30 items). A score of 5 and above is considered to have non-clinical implications (Frederiksen et al., 2022).

**Figure 1:**
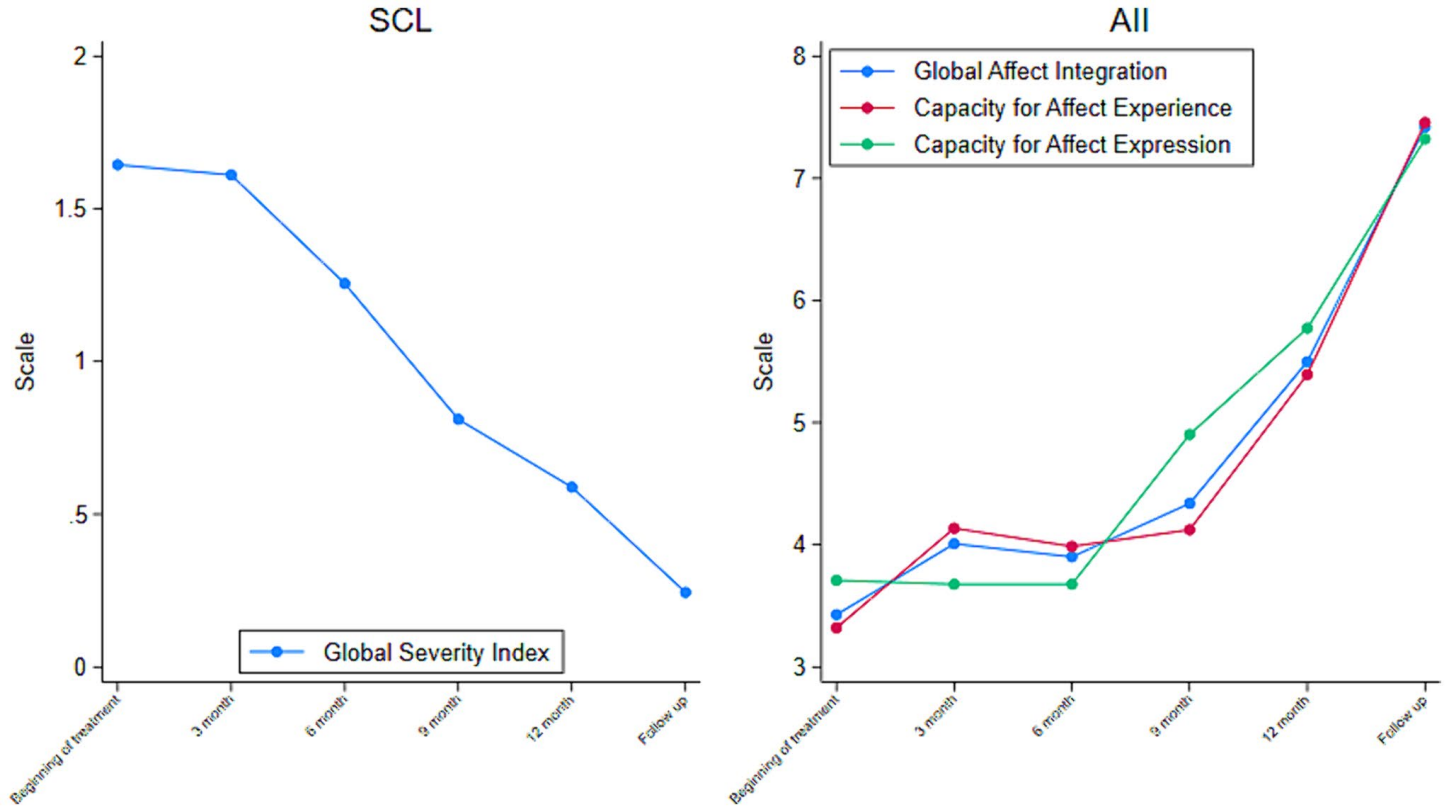
Outcome throughout treatment. Global Scores from the Hopkins Symptom Checklist (SCL-92) **(Left)** and the Affect Integration Interview (AII). Derived From the Quarterly Questionnaires Reported by a BPD Patient in Treatment (*N* = 1) **(Right)**.

The AII and SCL-92 indicated significant improvement throughout treatment. The AII scores showed a consecutive increase in affect integration (start: 3.43; end: 5.5; follow up: 7.42), capacity of affect expression (start: 3.71; end: 5.77; follow up: 7.32) and affect experience (start: 3.32; end: 5.40; follow up: 7.46) (Figure 1). The AII scores indicated the development of a more fluent and functional integration of affect in cognition, motivation, and behavior. The SCL-92 score was above the clinical cut-off at the beginning of therapy, indicating severe distress (GSI: 1.64) (Figure 1). At the end of treatment, the score was below the clinical cut-off comparable to a Danish norm group of a non-clinical population (GSI: 0.59, effect size: 1.06) (Olsen et al., 2006). Six months of follow-up showed persistent, significant improvement (GSI: 0.24).

The patient, anonymized as “Sophie” to protect confidentiality, was a woman in her middle 20s. She was initially assessed by a psychologist using the Present State Examination (Wing et al., 1974) and the Structural Clinical Interview for DSM-IV Axis II Personality Disorders (SCID II) (First, 2014). Inclusion criteria in the outpatient clinic were a primary diagnosis of personality disorder, and exclusion criteria were mental retardation, and active alcohol or drug abuse. Sophie was found to meet the criteria for BPD and Avoidant Personality Disorder. Prior to recruitment in the EPICLE project, she was given oral and written information about the study, and written consent was obtained from her and her therapist to participate. No payment or other compensation was given. She was informed that non-participation would not affect her treatment. Sophie was referred to the clinic because of severe interpersonal difficulties, emotional dysregulation, increased isolation, and decreased functionality in her everyday life. Her case formulation described how Sophie came from a home with a sister, a father who often lost his temper, and a mother with high demands who reacted with silence or criticism if Sophie did not live up to her expectations. As a child, Sophie often experienced angry outbursts or reacted to emotional distress by withdrawing and isolating herself. The family did not talk much about mental states or emotions at home, and Sophie often felt alone. In her teens, she started using alcohol and drugs when feeling lonely as a way of coping with emotional distress. She experienced her first depression at the age of 15. Most of her relationships were intense, conflictual, short-term, and unstable in this period. She described herself as promiscuous and as a person seeking attention from others through sexual activity. Her promiscuous behavior changed in her early 20’s while living in a longer relationship with an alcoholic, controlling, and devaluating partner. She started to self-harm and became suicidal with one major suicide attempt. She managed to end the relationship, and after this, her primary emotion regulation strategy became overregulating instead of acting out. Overregulation was still her primary way of coping when entering treatment.

Sophie was offered 1.5 years of mentalization-based therapy (MBT) for personality disorders, including individual therapy and group therapy. She received 1 year of individual therapy and 8 months of group therapy. She terminated the group early because she had moved to a new city. She had 34 individual sessions lasting from 45 to 64 min. The therapeutic work concerned engaging Sophie in a mentalizing process about her current pattern of withdrawal and learning new ways of understanding herself and others and managing her emotions. The therapist was a female psychologist in her early 30’s with 5 years of expertise using MBT. She had formal training and experience with MBT from the clinic and received monthly supervision from an expert in the field.

### 2.2 Data collection

#### 2.2.1 The rupture resolution rating system (3RS)

The observational Rupture and Resolution Rating System (3RS; Eubanks et al., 2019) was applied to assess rupture and repair processes across and within sessions. While watching 5-min segments of video-recorded sessions, coders noted events of tension in the therapeutic relationship characterized as either confrontation ruptures or withdrawal ruptures and resolution strategies. Sessions were coded using both video and audio material. Sessions were coded for either no salient rupture or resolution or a salient rupture or repair segment. The 3RS ratings were collected via Research Electronic Data Capture (REDCap) (Patridge and Bardyn, 2018). This study included coding 5-min segments of rupture and resolution of 28 sessions. Six sessions were not coded because of missing video material or technical issues (i.e., sound or picture problems).

#### 2.2.2 Physiological measures

HR was monitored for both patient and therapist using the photo plethysmogram (PPG) method to measure IP. Blood volume pulse (BVP) was monitored using PPG BioNomadix by attaching a pulse transducer to the index finger. Data were collected using the BioNomadix system of wearable devices at 125 Hz. Twenty-one sessions were available for HR data analysis, while 13 sessions were excluded due to electronic or measurement failures.

#### 2.2.3 Qualitative data collection

Data collected for the qualitative analysis consisted of video material, audio, transcriptions, basic clinical background information consisting of therapist’s notes, the patient’s case formulation, and condensed notes of clinical content made by the primary 3RS rater after rating each session. All sessions were video recorded with two AXIS P5514 PTZ Network Cameras, one 6x AXIS M1025 Network Camera, and audi was recorded with two Sennheiser MKE2 wireless microphones. Noldus Observer XT software was used to synchronize the recordings. Verbatim transcriptions based on Jefferson’s transcription system were performed (Jefferson, 2004).

### 2.3 Data analysis

**Figure 2:**
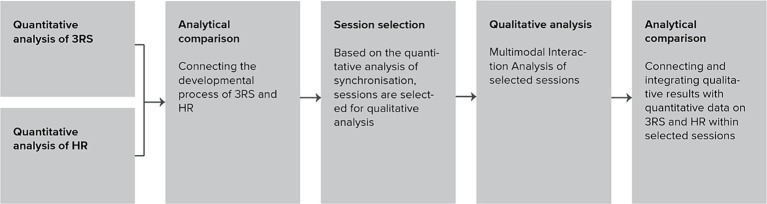
Analytical steps.

The first step of the analysis included a quantitative analysis of the 3RS ratings to answer RQ1 and a quantitative analysis of IP to answer RQ2. After the first two steps, an iterative, multi-layered analytical comparison of the quantitative analyses of IP and 3RS ratings was conducted and interpreted on the background of basic clinical information and process notes to answer RQ3. Finally, a qualitative analysis of the multimodal interaction between patient and therapist was performed on sessions selected based on the quantitative analysis of IP to give indication of within session dynamics to answer RQ4. The qualitative multimodal interaction analysis was interpreted on the background of data from the quantitative analysis of IP, transcripts, video recordings, audio material, and rupture and repair ratings. The analytical steps were each applied separately and blinded to each other before the findings were synthesized to form a multi-layered representation of the results and clinical process (Avdi and Seikkula, 2019; Figure 2).

#### 2.3.1 Quantitative analysis of rupture and resolution processes

To answer RQ1 and RQ3 concerning the development of rupture and repair over the course of treatment, the frequency of each rupture or resolution segment was summed up across all 5-min segments of each session. To answer RQ4, rupture and repair ratings were compared to physiological within session dynamics on a sentence level.

3RS has shown good interrater reliability (Eubanks et al., 2019) and given the exploratory nature of this study, consensual agreement was used to determine each code assigned to a segment. One primary rater coded the entire course of treatment, while four sessions were rated for consensus by three raters with excellent interrater agreement. Before rating the sessions, all three coders received approximately 50 h of theoretical background and training in the manual, including reading the manual, attending an introduction course, and practicing video-session coding. Additionally, all three coders met weekly and co-rated the video material and discussed discrepancies for 3 months until sufficient consensus was reached before rating this study’s data.

#### 2.3.2 Quantitative analysis of physiological measurements

Heart Rate was initially studied on the PPG signal data (from the RR output, referring to the interval between heart beats) to measure IP. RR distances were calculated and analyzed in each session using Recurrence Quantification Analysis (RQA) (Webber and Marwan, 2015).

RQA is a method of nonlinear data analysis for investigating dynamical systems. It quantifies the number and duration of recurrence of a dynamical system presented by its phase space trajectory. This technique is particularly useful for analyzing short and nonstationary data, where other methods might fail (Orsucci et al., 2006; Wallot et al., 2015). RQA is based on the construction of recurrence plots (RP), which are visual representations of the times when a state in a dynamical system recurs. The analysis provides several measures of complexity, such as recurrence rate (REC), determinism (DET), and entropy, among others (Webber and Marwan, 2015). These measures can reveal important characteristics of the dynamical system, such as predictability and stability. In this work we compared the REC of RR time series recorded from the patient and the therapist. RQA is a statistical and graphical tool used in various domains to analyze time series data, including physiology, engineering, chemistry, and earth sciences demonstrating its versatility and utility in understanding nonlinear dynamics and complex systems (Zimatore et al., 2021). RQA has proven useful for quantifying non-stationary coordinative emotion regulation patterns between patients and therapists during the therapeutic process, both throughout treatment and within sessions, which actualizes its use in the examination of how IP may relate to rupture and repairs across and within the treatment process (Fusaroli et al., 2014). The procedure to obtain the RQA measures is described in more detail in the Supplementary material and furtherly in Marwan and Webber (2014).

Two different procedures were applied to answer the overall aim of this study: the Cross-RQA techniques and the RQA epoch-by-epoch (RQE). The Cross-RQA was applied to answer RQ2 and RQ3. It has proven able to detect periods in the clinical process with either significant patterns of emotional connection/synchronization or dysregulation between patient and therapist, which are found to be periods especially important concerning either strengthening or challenging the alliance and could, as such potentially reflect periods of ruptures and repairs (Kodama et al., 2018). Cross-RQA involves the application of RQA to compare two or more time series simultaneously (here, patient and therapist: 1 epoch of 20,000 points on two time series) (Fusaroli et al., 2014; Schultz et al., 2015).

To answer RQ4, the RQE technique was used. RQE allows to examine how the recurrence properties of the HR of the patient and therapist change or evolve over time, potentially revealing dynamic patterns, transitions, or other temporal characteristics of implicit emotion regulation processes on a moment-to-moment basis which makes it especially interesting to compare with verbal and observable processes within sessions to get a more in-depth understanding of the correspondence between implicit and explicit alliance developments. The whole time series are divided into epochs. Each epoch represents a specific time window or interval (1 epoch of 2,400 points (corresponding to 5 min) were shifted by 240 points). The percentage of recurrence points (REC) for the patient and the therapist during sessions were examined separately.

Sessions with higher synchronization (corresponding to higher value of REC, found in the Cross-RQA) were included for the within-sessions analysis. RQE (RQA epoch-by-epoch) was applied to identify sequences of HR recurrence patterns for the patient and therapist within the sessions.

#### 2.3.3 Qualitative analysis of the therapeutic interaction within session

A qualitative multimodal interaction analysis was applied on the identified RQE session sequences to examine RQ4. Relying on the work of Charles Goodwin, multimodal interaction analysis focuses on micro-segments and can provide systematic in depth knowledge of the therapeutic process using transcript material from video and/or audio recordings (Goodwin, 2009). It involves taking an embodied perspective when performing highly detailed descriptions of the participants’ interaction, including verbal utterance, gestures, head-and body movements, and gaze (Goodwin and Bjørndahl, 2018). It focuses on *how* the interaction takes place rather than *why* it takes place (Muntigl and Horvath, 2024). Multimodal interaction analysis has proven productive in the examination of both explicit and implicit processes of therapeutic interactions (Davidsen and Fosgerau, 2015). A particular focus of attention is put towards the cooperative actions examining how interacting partners use and transform embodied behavior to create new joint meaningful actions (Goodwin, 2000; Trasmundi and Philipsen, 2020).

The analytical procedure was conducted as a detailed examination of what happened during the interaction, followed by determining interactional patterns in the sequences. ELAN annotation software (Software, 2023; Wittenburg et al., 2006) was used to secure a systematic exploration. Each sequence was interpreted relying on the analyst’s reflexivity and evaluation of the results gained from the analytical process. To ensure reflexivity, two researchers conducted the analysis, one of them a specialist in the field of interaction analysis. One researcher did the primary analysis, while the other did a critical evaluation of the transcripts, observations, arguments, and interpretations. Each analysis was challenged through an iterative process until interrater agreement and adequate reliable results were achieved.

After conducting the multimodal interaction analysis, the results were interpreted on the background of the RQE analysis and 3RS ratings using the same iterative procedure as applied in the multimodal interaction analysis.

## 3 Results

### 3.1 RQ 1: How does rupture and repair processes develop over the course of treatment?

**Figure 3:**
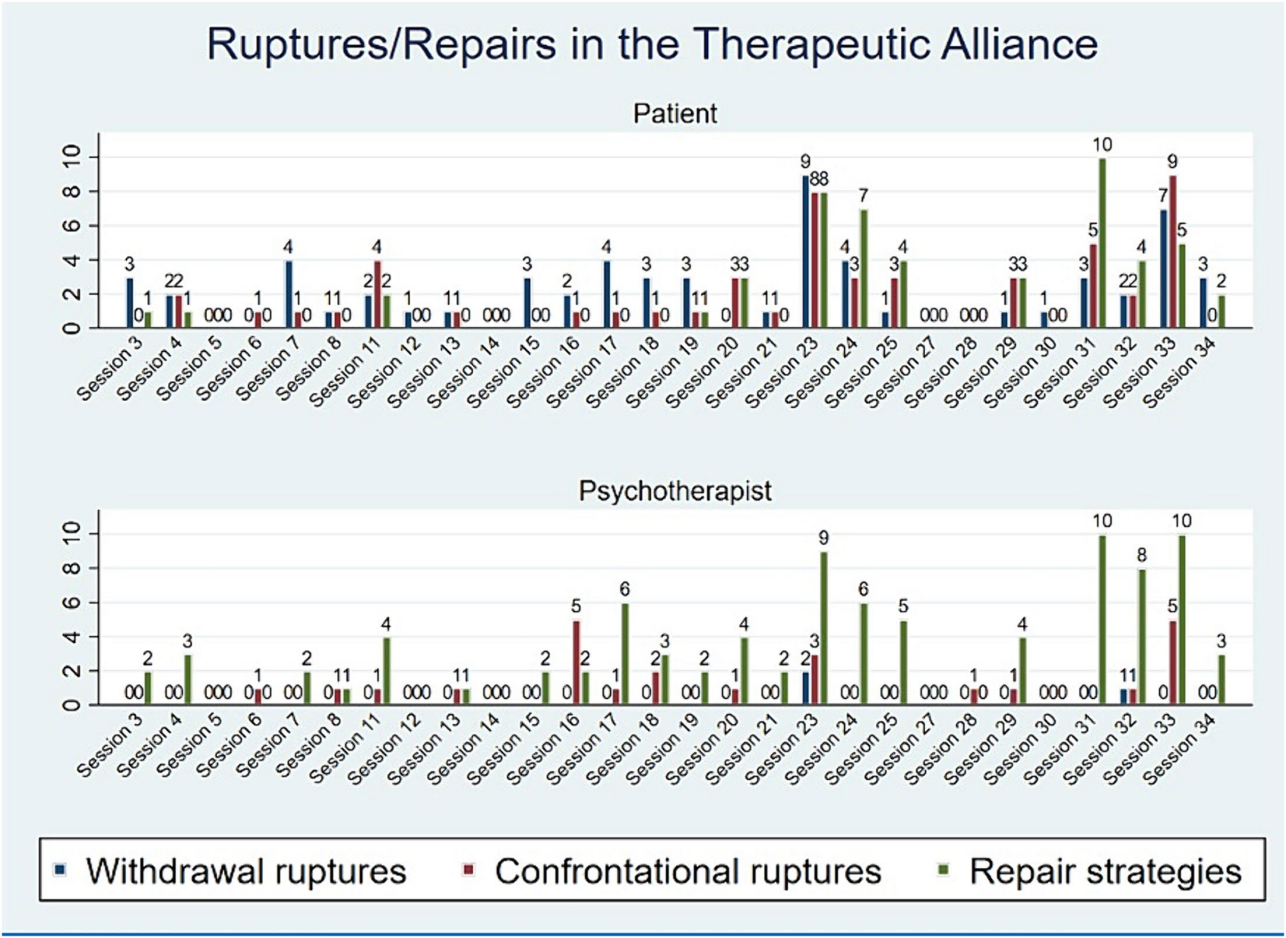
Rupture and repair ratings over the course of treatment for the patient and psychotherapist.

The results in relation to RQ1 revealed that ruptures were present in almost every session, whit only 3 sessions not including ratings of rupture segments. Of the 28 sessions rated for rupture and repair segments, 51 confrontation rupture segments were identified for the patient, and 24 for the therapist. Sixty-one withdrawal rupture segments were identified for the patient, while three for the therapist. Fifty-one repair segments were identified for the patient, and 89 segments for the therapist. A large peak of rupture segments was rated in session 23 and session 33. These sessions were surrounded by a noteworthy co-occurrence in the use of repair strategies in the last part of the treatment process (Figure 3).

### 3.2 RQ 2: How does IP develop throughout treatment?

**Figure 4:**
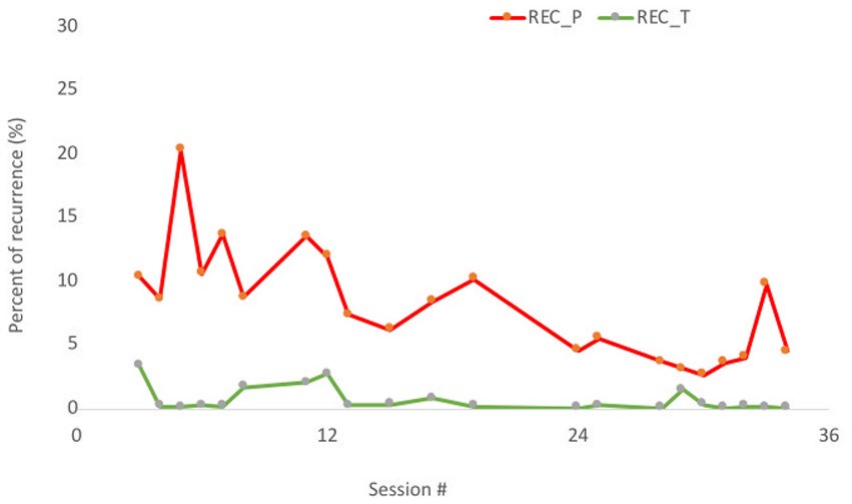
Percentage of recurrence (REC) in 21 sessions. Patient = red line (dark gray in the printed version); therapist = green line (light gray in the printed version).

To answer RQ2 an RP was obtained (1 epoch with 20,000 points) for every session, and the percentage of recurrence (REC) was obtained on the whole time series (about 20,000 points, 1 h = 28,800) for the patient and therapist independently (an overview and interpretation of the REC independently for the patient is represented in the Supplementary material). The Pearson correlation between REC_T (therapist) and REC_P (patient) was high (*r* = 0.61) (Figure 4).

**Figure 5:**
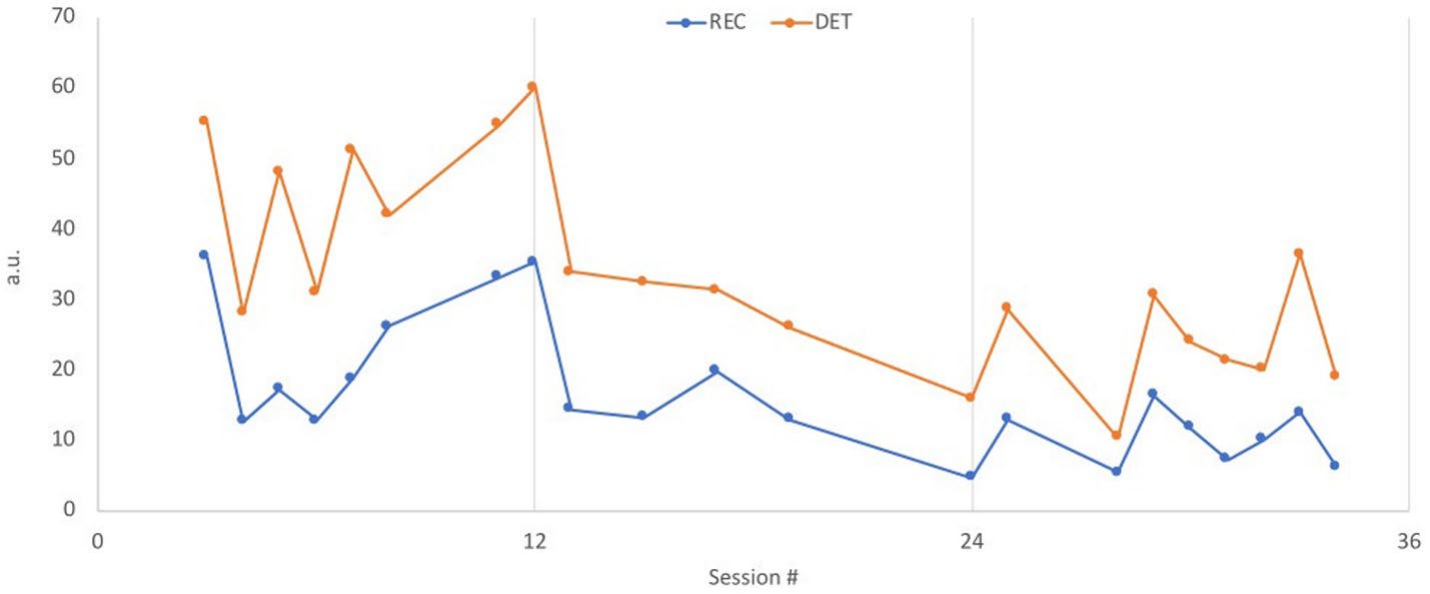
CRQA, cross recurrence in therapist-patient dyad. The two measures REC and DET are reported for every session; REC = the blue line (dark gray in the printed version) is the percentage of recurrence points; DET = orange line (light gray in the printed version) is a measure of the longest diagonal lines, indicating the longest recurrence periods in each session.

After conducting the RQA analysis separately for the patient and the therapist, a Cross-RQA analysis for each patient-therapist pair in each session was performed to examine IP patterns throughout treatment. The Cross-RQA analysis revealed how the entire course of therapy seemed to be divided into three phases (Figure 5): sessions 1–12: initial adaptation; sessions 13–23: stability; and sessions 24–34: new oscillations.

### 3.3 RQ3: How does IP reflect rupture repair processes over the course of treatment?

The results in relation to RQ3 showed, how the initial phase of treatment (sessions 1–12) revealed the fewest rupture repair segments. The therapeutic work concerned developing a case formulation and collaborating on a common understanding of Sophie’s difficulties. In the middle phase, withdrawal and confrontation segments increased (sessions 13–23). In this period the therapist and Sophie started working more intensively with Sophie’s pattern of trying to adapt to her mother’s demands by withdrawing and suppressing her own reactions, often resulting in increased shame and self-hatred. The identification and negotiation of this pattern and the consequences of their interpersonal dynamics was conflicted for her. Her initial aim in the treatment was to be better able to amend the mother’s demands, and the therapeutic process of negotiating the goals and tasks of treatment could reflect the increased number of ruptures in this phase. Additionally, the middle phase was characterized by treatment instability, with two longer periods without therapy (both times approximately 30 days, after sessions 13 and 16), which could also reflect the increased rupture segments. Despite the increase in ruptures, Sophie experienced progress during the middle phase, which is reflected in the outcome measures. She started working with her experiences of often feeling alone and abandoned, especially by her mother, leading to a better capability of expressing her feelings towards the mother and setting proper boundaries in their relationship. In the last identified phase (sessions 24–34), withdrawal and confrontation segments were almost as prevalent as in the middle phase, while repair segments for both the therapist and Sophie increased significantly. In this phase Sophie expressed increased concern about ending the individual therapy. She got angry at the therapist for the periods without therapy where she had felt abandoned and alone. Sophie and her therapist started a process of exploring their relationship more intensively and the parallels between Sophie’s experience of feeling abandoned by the therapist, and experiences of having felt abandoned by her mother, as well as in other relationships. The new Cross-RQA patterns in this period could reflect their mutual increase in resoultion ratings and the intense work in their relationship. At the end of treatment, she stated that she had gained more confidence and trust in both herself and others when facing difficulties. When she lost control or withdrew from discussions, she managed to go back and talk to the person involved and came away with new experiences of repairing relationships.

### 3.4 RQ4: How does IP reflect the multimodal therapeutic interaction within sessions?

In relation to RQ4, 13 segments with higher REC in the HR time series were identified in the RQE analysis within the four sessions derived from the Cross-RQA analysis. In five of the identified segments mixed rutpures (segments including both withidrawal and confrontation) and repair ratings were represented in the 3RS ratings, two segments included withdrawal ratings, and six segments were identified with no subtle rupture repair ratings. Below, three segments from sessions 3, 5, and 33 are presented to illustrate the interactional process and the findings regarding how HR REC may underline the clinical process. The first two segments are from the initial phase of the treatment process and concerns Sophie’s primary pattern of withdrawal, while the last segment is from the third phase of the treatment process showing exploration of ruptures occurring in the relationship between Sophie and the therapist.

#### 3.4.1 Session 3: Withdrawal

**Figure 6:**
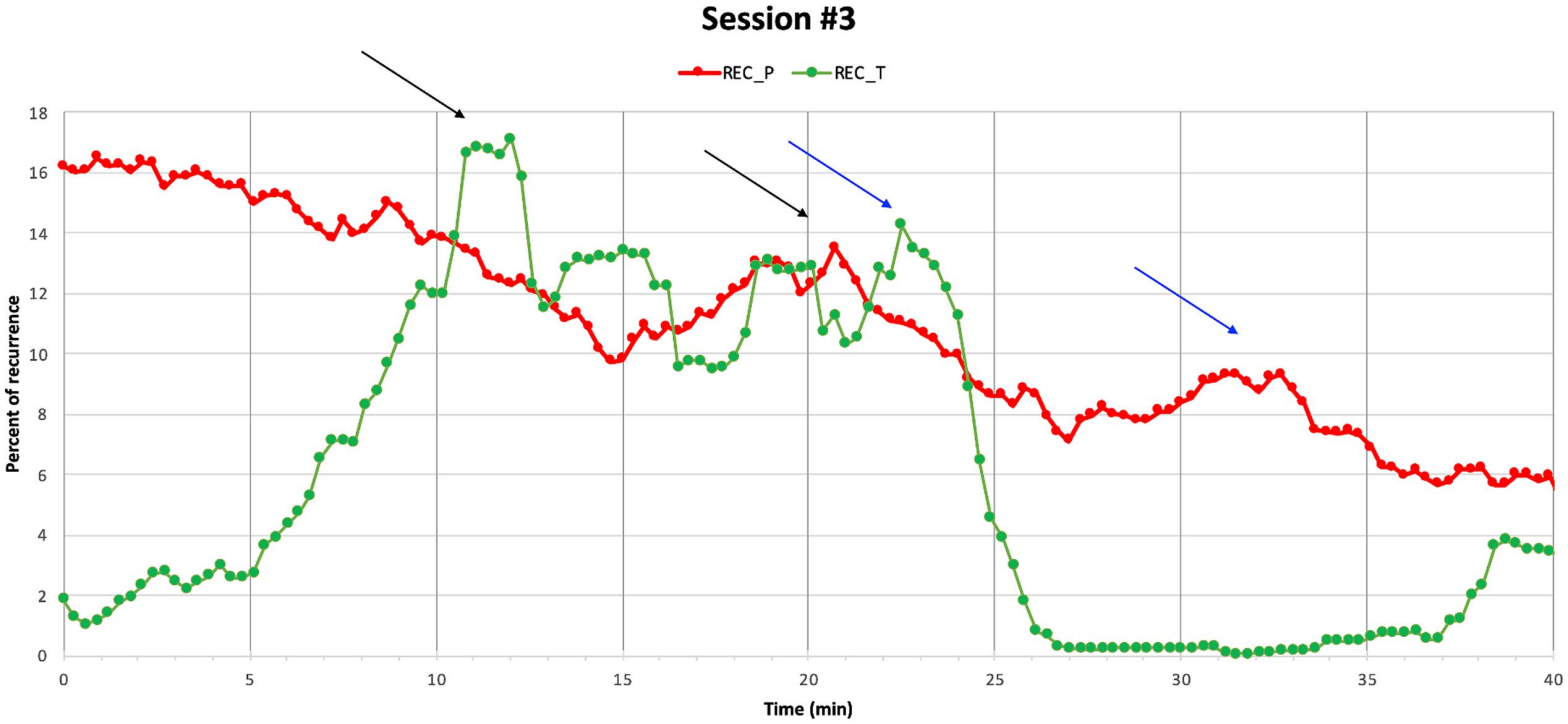
Session 3. A peak in Sophie’s REC around minute 20 might be connected to a peak around minute 10 where REC solicitation for the therapist is observed. In minute 23 there was a peak in the therapist’s REC, which might relate to minute 33, where a peak in the REC of Sophie was identified.

In session 3, Sophie tells the therapist that her grandmother will probably die soon as she is very ill. Sophie has not talked to anyone about her emotional reactions and tries to deal with her sadness by isolation and by distracting herself. The rupture repair ratings in this segment primarily indicate Sophie’s withdrawal. In this segment a peak in the therapist’s REC parameters is observed at minute 10 (see (Figure 6)).

**Minute 10: mirroring sadness**

P: so (0.6) it was it was a really hard blow for all of us as (0.4) I still haven’t quite figured out how to deal with it

T: mm (0.4) no

P: no

T: but I can see that it makes you sad

P: yes hehhh yes it does .hhh

T: yes

P: uhmm because she is the driving force of so many things

T: mm

P: uh (1.1) and among my grandparents she is the one I have the closest relationship with

The sequence starts by Sophie saying, “it was a really hard blow to us all,” referring to finding out about the grandmother’s illness. She continues explaining that she does not know how to deal with it. Instead of moving along with Sophie’s focus on what to do, the therapist takes a turn, and focuses the attention on Sophie’s affective state of mind, responding, “but I can see that it makes you sad.” This intervention from the therapist may be conceptualized in terms of what Ekberg et al. (2016) call *emotional inference*, referring to how a therapist formulates a mental state marked as an inference using an evidential verb. Emotional inference has been found to be used when a patient’s reply is affective but does not involve explicit descriptions of emotions. It has been identified as an empathic response that facilitates exploration and verbalization of affect (Peräkylä, 2019). As Sophie begins the sentence by saying, “it was a hard blow,” she is implying that the information was difficult without verbalizing her affect directly. The therapist’s response that she *can see* it makes her sad, indicates assurance of how Sophie is feeling based on the therapist’s observations. The marked response from the therapist is followed by Sophie’s confirmation and elaboration, while laughing, and tears start running from her eyes. The adequate mirroring seems to facilitate enough security for Sophie to get in contact with her sadness as she starts to cry followed by an inhale and an elaboration, where Sophie continues talking about her relationship with the grandmother. At the same time, Sophie’s laughing indicates emotional distress when mirrored by the therapist, reflecting part avoidance of her sadness.

In this sequence, the therapist’s physiological attunement seems to underline her explicit empathic responding. This therapeutic multimodal action is followed by Sophie allowing the therapist to see her vulnerability. As Sophie tends to deal with her emotions alone, this is most likely a new and insecure experience for her. The correspondence between the therapist’s increased REC and verbalization of Sophie’s affect might as such facilitate a process of enough security for Sophie to engage in sharing her emotions instead of fully withdrawing, which has often left her feeling alone at difficult times in her life.

#### 3.4.2 Session 5: Non-rupture segment

**Figure 7:**
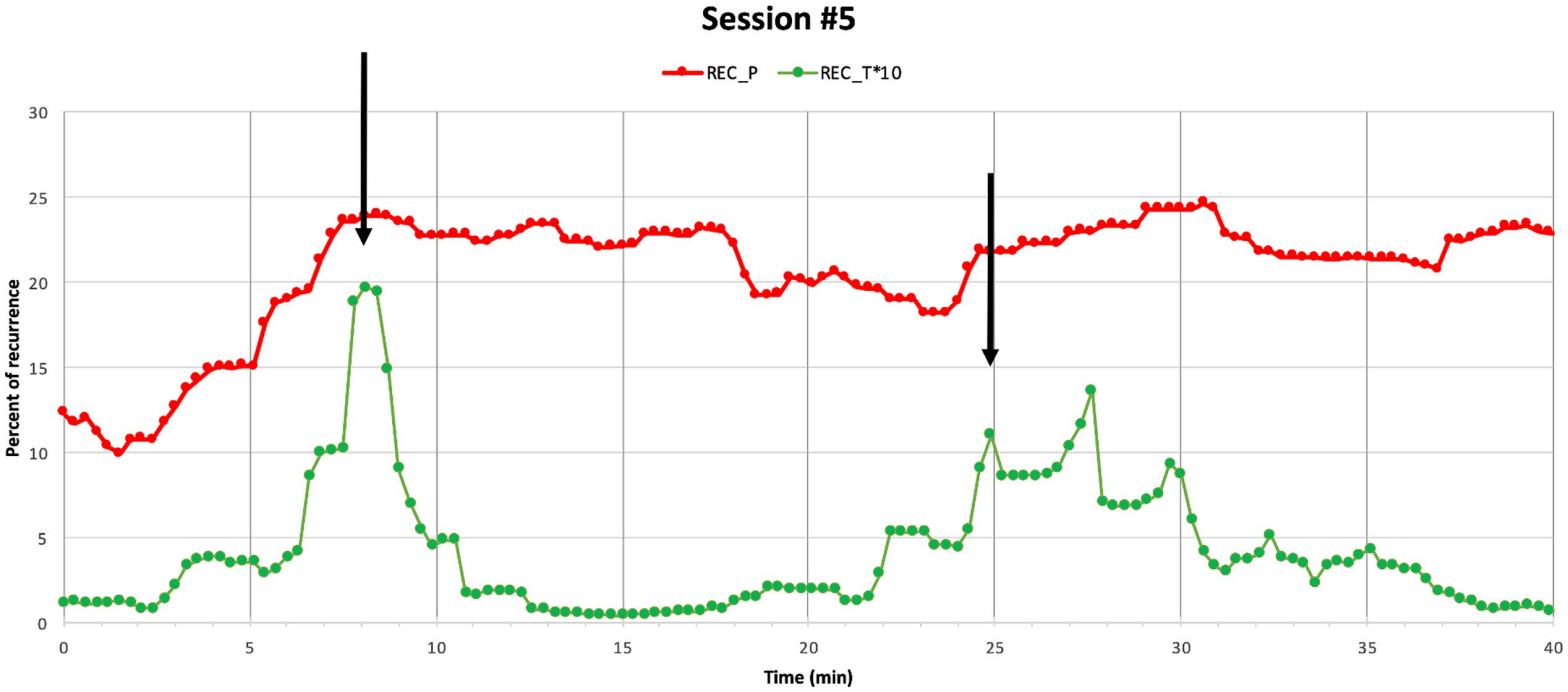
Session 5. Percentage of recurrence of Sophie’s (REC_P) and her therapist’s (REC_T*10) HR time series within Session 5. Black arrows show simultaneous peaks. Note that REC the therapist’s HR time is presented in an enlarged scale (*10).

In session 5, Sophie tells the therapist that she has talked to an old friend—a friend whom she was afraid of facing, as she had often been acting out on her during their friendship. Initially, she describes how she wanted to make amends, but at the same time she was afraid of being rejected and had experienced an impulse to avoid confrontation. Interestingly, session 5 reveals how the REC parameters are observed to synchronize between the patient and the therapist two times across the session (Figure 7), but no subtle rupture or repair ratings are identified in these segments. The first segment of corresponding REC between Sophie and her therapist is presented below.

**Minute 8: brave**

T: wow how brave you have been

P: yes ha:ha:

T: I think

P: It [aeh]

T: [actually]

P: It was a big day

T: So you did (0.6) just to highlight it a bit

P: yes

T: you did something different than barricading yourself

P: yes

T: and hide behind your wall of protection

P: yes

T: right

P: yes

T: you did something else (0.3) than you usually do

P: yes

T: Than you would have done

P: yes (0.6) i:t uhh (2.5) so that that having somewhere the strength to go even if (0.6) the risk existed that

T: yes

P: that I would just be rejected that eh (1.4) that was huge

The therapist opens this sequence with a *highlighting formulation*, acknowledging how brave Sophie has been by referring to a previous turn in the session where Sophie told her that she did not withdraw but talked to her former friend about their past resulting in repairing their relationship. In interactional analysis, formulations have been described as statements transforming the patient’s talk into psychological subjects suitable for therapeutic work and sense-making. They can be used to highlight an agenda and prepare the patient’s attention for subsequent actions from the therapist (Antaki, 2008). In this segment the highlighting formulation draws the attention of Sophie to *how brave she had been* and prepares her for exploring her experience. The therapist’s initial acknowledgement is followed by an array of encouraging statements (“you did something different than barricading yourself,” “and hide behind your wall of protection,” “right,” “you did something other than you usually do,” and “that you would have done”). “The statements are mainly confirmed by Sophie up until a point where she explicitly acknowledges, reflects, and elaborates on what she has done, saying it was huge, as she knew there was a risk of being rejected. As the therapist’s statements are clearly referring to Sophie’s accomplishment, it is possible that this paves the way for Sophie to use the acknowledgement as a resource to take agency and consolidate the accomplishment as her own (Weiste and Peräkylä, 2013).

The corresponding HR REC between the therapist and Sophie could indicate a shared emotional experience and relatedness, where Sophie feels herself reflected in the therapist’s verbal and nonverbal responses leading to a process where Sophie engages in the therapeutic work by reflecting and elaborating on her progress. The segment indicates that IP seems not only to be present while experiencing ruptures, but also while sharing important emotional moments of improvement.

#### 3.4.3 Session 33: Mixed rupture repair

**Figure 8:**
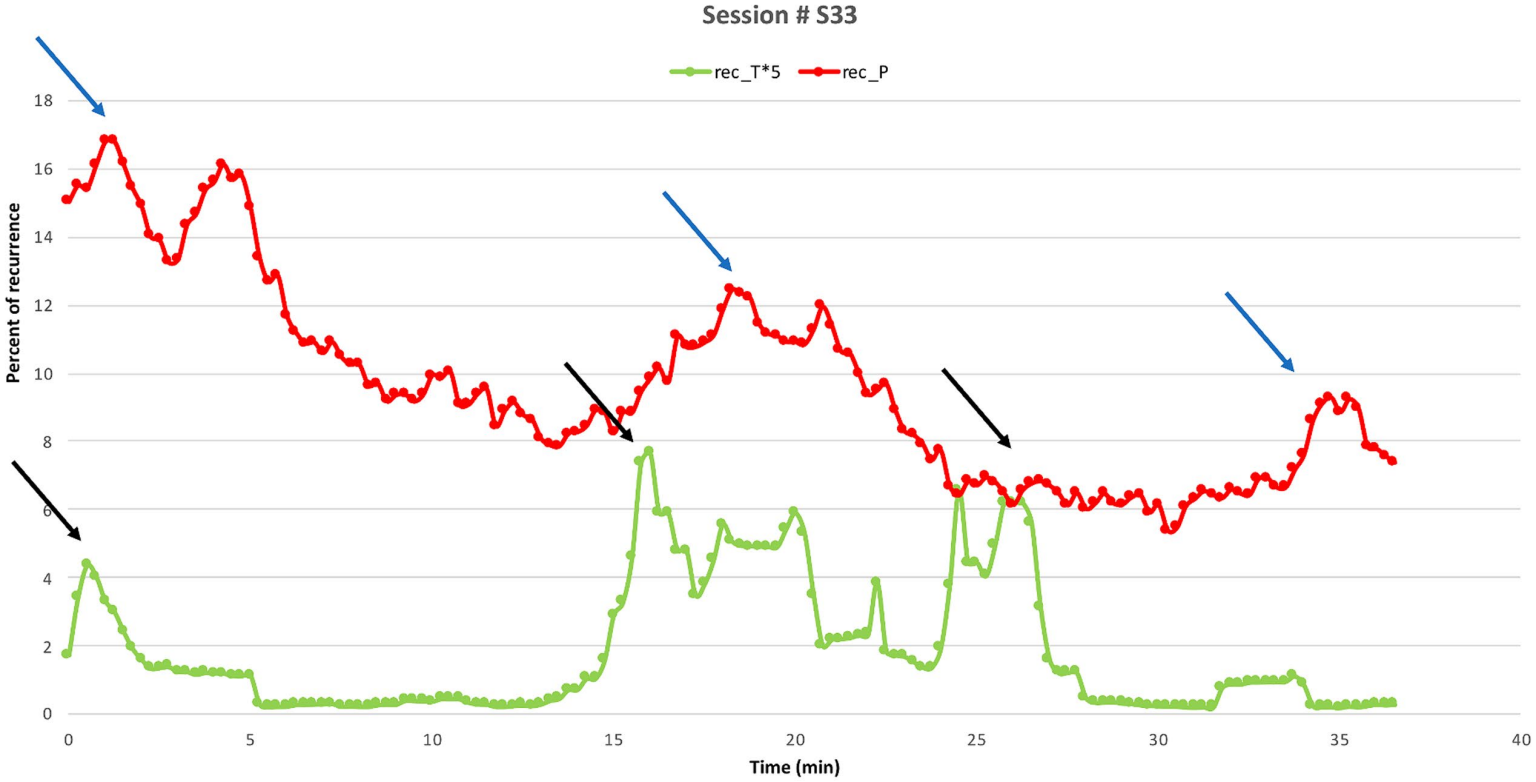
Session 33. Percentage of recurrence of Sophie’s (REC_P, red line) and her therapist’s (REC_T*5, green line) HR time series within Session 33. The peaks on the therapist’s lines (black arrows) precede those on Sophie’s (blue arrows) by approximately 1 min (equivalent to 3 epochs). The three peaks occurred at the following times, respectively: the first one (0.60 min T, 1.50 min P); the second one (19.20 min T, 20.10 min P); and third one (24.0 min T, 24.54 min P). Note that REC of the therapist’s HR time is presented in an enlarged scale (*5).

In session 33 an overlap between maximum corresponding HR REC between Sophie and her therapist and maximum rupture repair is observed. In this session Sophie is angry with the therapist for not taking her perspective in group therapy, which left her feeling alone and betrayed and made her lose emotional control during group resulting in an angry outburst. During the session a significant HR REC correspondence is observed three times with approximately one minute between the peak of the therapist’s HR REC followed by a peak in Sophie’s HR REC. In the following a description of the first segment is provided (Figure 8).

**Minute 1-2: Addressing a rupture**

T: yes (1.8) but maybe there is something unfinished (0.2) from group or what

P: hhh yes heh:heh[heh]

T: [yes]

P: [yes]

T: [I] am not supposed to define what we are going to talk about today=

P: [no]

T: =[it] may not be important to you=

P: uh it [is]

T: =[but] I thought it might be

P: yes

T: [yes]

P: [I uh] hhh uh I I left with a feeling of shame for losing control which I really did not like=

T: [okay]

P: =[uhm] I think I ident- so I both identified- and it really really hurt that (0.8) the way what I had said was reversed

T: yes

P: and used

T: yes

P: and especially that (0.1) you said it really really hurt

T: yes

P: uh (0.7) and then I like I’ve lost control in front of someone I don’t actually trust (0.7) right now (.) in front of Tina

T: oh in front of Tina

P: yes uh I did not like it

T: no (0.6) like that

P: yes

In this extract, the therapist starts by addressing the group therapy session with a question: “but maybe there is something unfinished (0.2) from group or what,” inviting Sophie to explore the rupture that occurred between them during group. Sophie engages in the exploration in a confronting manner and responds with a “.hhh yes” with emphasis on “yes” followed by laughter, while gazing directly at the therapist, both signaling the seriousness of the subject by her marked “yes” and gaze, and at the same time withdrawing by laughing. The therapist responds by retreating saying that it is not her decision what they should talk about. Sophie confirms with an overlapping “no” and the therapist expands her turn by saying that she does not know whether group has been on Sophie’s mind but continues saying she thinks has been while nodding and looking Sophie in the eyes. In interaction analysis, Muntigl et al. (2013) describe the term *retreat*, referring to the therapist withholding from responding verbally while awaiting the patient’s elaboration of his/her perspective. Retreat is found to be associated with the therapist making non-verbal displays of nodding, which functions as affiliations and validations of the patient’s position (Stivers, 2008). In this segment, the therapist frames an important topic, guiding the attention of Sophie followed by the therapist’s verbal retreat while nodding at Sophie, which seems to leave space for Sophie to take control and decide how to proceed. This therapeutic multimodal action seems to foster, that the therapist manages to retain an empathic and emotional relatedness with Sophie, which leaves space for Sophie to move the conversation forward and gives the opportunity to explore their divergent and/or similar perspectives (Muntigl et al., 2013). This interpretation is reflected in Sophie’s next turn, where she engages in elaboration and directly confronts the therapist stating how the therapist’s words in the group therapy session really hurt her. Throughout the rest of the sequence, the therapist mainly responds with explicit nonverbal displays of nodding and explicit confirmations indicating engagement and understanding letting Sophie elaborate her perspective further (Stivers, 2008). At the end of this sequence the therapist says: “oh in front of Tina,” repeating the last part of what Sophie just said, but adds an “oh,” and makes a deep nod, signaling affiliation and new understanding of Sophie’s perspective (Heritage, 1984).

During this rupture repair segment, the therapist’s increased HR REC corresponds to her intervention addressing the rupture from group. The therapist’s increased HR REC might reflect her intuition that this topic is a potentially high-risk intervention which is likely to increase tension between them. As Sophie engages in the elaboration of her point of view in a confronting manner this correlates with a peak in her HR REC that follows that of the therapist. The therapeutic strategy of retreat, confirmation and nonverbal displays of nodding endorses and foregrounds Sophie’s perspective which seems to help facilitate a process of offering Sophie safety to attune and confront her upsetting experience in their relationship and move forward working through the rupture (Muntigl, 2020; Muntigl and Horvath, 2024). Thus, the active retreat leaves space for Sophie’s conflicting perspective from group and gives the opportunity for the therapist to orient towards their disagreement as a challenge of intersubjectivity in need of repair and seems to facilitate collaboration and elaboration (Muntigl, 2020).

As exemplified in these three segments the analytical comparison and interpretation of HR REC, 3RS, and interactional dynamics revealed how HR REC reflected important alliance processes when facing difficulties as well as when sharing improvement. Analyzing all 13 segments using the same iterative procedure, the qualitative analysis reveals how increased HR REC seems to underline alliance processes of emotion regulation, empathy, security, shared emotional experience, validation, sense-making, consolidation, and elaboration in correspondence with verbal markers of emotional inference, different types of formulations, epistemic markers, confirmation, and observable non-verbal markers including nods, gazes, smile, and laughter (an overview of all segments is available in Table 1).

**Table 1:** Multimodal interaction analysis.

Session	Segment	Recurrence measure in HR	Verbal and nonverbal cues	IP underlining the following alliance processes	Rupture/repair
3*	Minute 10	Therapist (T) increased REC showing potential correspondence to Sophie’s (S) increased REC in minute 20	Therapists use of emotional inference	Empathy Shared emotional experience Security	Withdrawal
3	Minute 20	S’s increased REC showing potential correspondence to T’s increased REC in minute 10	Overlapping talk Therapists use of continuers	Sense-making	None
3	Minute 23	T’s increased REC showing potential correspondence to S’s increased REC in minute 33	Therapists use of emotional inference	Empathy	None
3	Minute 33	S’s increased REC showing potential correspondence to T’s increased REC in minute 23	Therapists use of emotional inference and continuers	Empathy Shared emotional experience Security	Withdrawal
5*	Minute 8	Simultaneous increased REC responses in T and S	Therapists initiated shared laughter and use of highlighting formulations	Validation Affiliation Consolidation	None
5	Minute 25	Simultaneous increased REC responses in T and S	Therapists use of deep nods and continuers	Empathy	None
29	Minute 2.5	T’s increased REC showing potential correspondence to S’s increased REC in minute 20	Therapists use of continuers and summarizing	Empathy	None
29	Minute 13	T’s increased REC showing potential correspondence to S’s increased REC in minute 27	Therapists use of continuers	Empathy	None
29	Minute 20	S’s increased REC showing potential correspondence to T’s increased REC in minute 2.5	Therapists use of rephrasing formulations	Empathy Sense-making Validation	Confrontation, withdrawal, repair
29	Minute 27	S’s increased REC showing potential correspondence to T’s increased REC in minute 13	Therapists use of relocating formulations	Sense-making	Confrontation, withdrawal, repair
33*	Minute 0.5	Lagged increased REC with precisely 1 min between peaks in S’s and T’s HR response	Therapists use of retreat, deep nods and continuers	Empathy Validation Emotion regulation Affiliation Security	Confrontation, withdrawal, repair
33	Minute 19	Lagged increased REC with precisely 1 min between peaks in S’s and T’s HR response	Patient’s initiated laughter	Emotion regulation	Confrontation, withdrawal, repair
33	Minute 24	Lagged increased REC with precisely 1 min between peaks in S’s and T’s HR response	Therapist initiated laughter	Shared emotional experience Sense-making Validation Security	Confrontation, withdrawal, repair

The selected segments (*) for representation in the multimodal interaction analysis.

## 4 Discussion

In this proof-of-concept exploratory single case study, we used a multimethod approach in analyzing the full course of treatment and within-session dynamics of a patient suffering from BPD. The purpose was to contribute to the field of alliance research both on theoretical, clinical, and methodological levels specifically by examining IP’s potential correspondence with alliance rupture and repair processes, and the clinical process.

### 4.1 RQ1. Rupture and repairs throughout treatment

In relation to RQ1 alliance ruptures were found to occur in 91% of the sessions including both ratings from the patient and the therapist. Even though differences have been identified according to the frequency of rupture occurrence in BPD treatment (Dotta et al., 2020; Gersh et al., 2017), this finding reinforces the majority of previous results showing frequent rupture occurrence in BPD treatment when measured with an observer based rating system (Cash et al., 2014; Muran et al., 2009).

Overall, withdrawal ruptures were identified to be more frequently than confrontation ruptures for the patient. This aligns with prior study findings and could reflect a more general pattern according to rupture occurrence in BPD treatment (Dotta et al., 2020; Schenk et al., 2021). However, the specific rupture ratings might also reflect the pathology of the patient in this study, which has also previously been proposed (Schenk et al., 2021). When entering treatment, the patient’s primary coping strategy when facing emotional distress in interpersonal relationships was avoidance, and it is likely to assume that this coping strategy is also reflected in the higher number of withdrawal ratings compared to confrontation ratings. While no conclusions can be drawn from this single case, future studies could include larger samples sizes to increase our knowledge of specific rupture types in BPD treatment in relation to pathological patterns, which has the potential to help guide the training and supervision of psychotherapists in the future.

The therapist’s contribution to the occurrence of confrontation ruptures were identified more frequent than contribution to withdrawal ruptures. Prior results primarily highlight the negative impact of the therapist causing ruptures, and have shown how the therapist’s contribution to ruptures is predictive of treatment drop out (Eubanks et al., 2018). In this study the therapist’s contribution to confrontation ruptures was found to increase in the last phase of the treatment process with two significant rupture peaks in session 23 and 33. Interestingly, repair was also increasing for both the therapist and patient in this phase. An explanation in relation to these findings might be that the therapist’s contribution to confrontation ruptures did in fact influence the alliance negatively during the clinical process especially in the final phase by underestimating or by being unaware of the interactional dynamics playing out during sessions (Ackerman and Hilsenroth, 2001; Colli and Lingiardi, 2009). The increased repair ratings in this phase could as such reflect increased mutual commitment and contribution to take responsibility to restore collaboration when facing tension. This interpretation is particularly interesting in relation to the patient, as the patient’s enlarged repair ratings could indicate progression according to increased commitment repairing ruptures across time. As previously outlined, patients with BPD show decreased commitment repairing tension, as they fear rejection or abandonment (Michael et al., 2021). Thus, the patient’s increased repair ratings could suggest increased trust and security addressing and working through ruptures across the treatment process. An additional possible explanation is that the therapist’s contributions to ruptures are not only causing negative influence on the alliance, but could also be related to the therapist’s more active position in MBT where addressing or contributing to ruptures might sometimes be a therapeutic mean of action to accomplish specific goals or to move the therapeutic work forward (Folmo et al., 2019; Folmo et al., 2021). Future studies could further examine the specifics of the interactional mutual collaboration in relation to the clinical impact and influence of the therapist’s and patient’s contribution to rupture and repairs to increase knowledge in this regard.

### 4.2 RQ2: The developmental process of IP over the course of treatment

According to RQ2 concerning the developmental process of IP throughout the treatment process, the Cross-RQA analysis showed indication of three phases with different HR patterns: initial adaptation (sessions 1–12), stability (sessions 13–24), and new oscillations (sessions 25–34). The phases identified in this case were found to reflect the therapeutic process where the initial phase was characterized by working with the patient’s case formulation, the middle phase was characterized by working with the patient’s primary pattern of withdrawal, and the final phase was characterized by working with the termination of the individual treatment, including an increased attention on the relationship between the therapist and patient in the therapeutic process. The identification of three phases aligns with previous descriptions of phases in psychodynamic treatment models consisting of: (1) an engagement/assessment phase including identification of typical, dominant, and recurring interpersonal patterns, a middle phase described by a working phase including a continuous attention on developing new understandings, making psychological sense of self and others, and developing new adaptive ways of resolving interpersonal difficulties, and an ending phase including an exploration of the patient’s experience of ending therapy, reviewing progress, and anticipating future vulnerabilities (Lemma et al., 2010, 2011). Thus, the results disclose the potential of applying Cross-RQA as a tool able to detect periods of different IP patterns and help locate crucial shifts in the therapeutic process. Additionally, Cross-RQA showed the potential as a tool able to identify sessions of maximum synchronization between patient and therapist. Compared to prior studies, increased interpersonal coordination in different modalities has previously been hypothesized as a tool capable of detecting rupture repair sequences, having identified associations between increased physiological arousal, synchronization, and rupture repair episodes (DiMascio et al., 1957; Mylona and Avdi, 2021). In this study, sessions with maximum synchronization were found to reflect sessions with both maximum rupture and repair dynamics, and sessions with no subtle rupture and repair. Also, in the within session analysis, the RQE analyses revealed increased synchronization in segments with and without subtle rupture repair ratings. This indicates, that the RQA analysis on the output of HR represented in this study may not be applied as a tool able to identify rupture repair sessions or segments as they are operationalized in the 3RS. Yet, the results strongly demonstrate that RQA is a tool able to capture important alliance processes that can help disclose key alliance features during challenging moments of interaction by revealing regulatory processes between interacting partners of either synchronization or desynchronization across and within sessions (Kodama et al., 2018).

### 4.3 RQ3: Analytical comparison of the developmental process of rupture and repair and IP over the course of treatment

In relation to RQ3 shifts in synchronization patterns were found to reflect significant aspects of the therapeutic work and process. The initial phase showed the lowest level of rupture repair ratings, while both confrontation ruptures and withdrawal ruptures increased in the middle and final phases, and repair strategies were found to be highest in Phase 3. The enlarged fluctuation of rupture repair segments and especially the increasing repair ratings in Phase 3 in this study might suggest increased affectivity and productivity in the therapeutic interaction as also proposed by Gersh et al. (2017). Hence, rupture repair processes emerging during the final phase of therapy might be seen as opportunities for therapeutic transformation, which also seems to be reflected in the new oscillations identified in the physiological responses. The rupture instances in Phase 3 could be viewed as new opportunities for corrective experiences if navigated adeptly with enough emotional security within the therapeutic interaction. This interpretation is reinforced when comparing the findings to the outcome data, where the symptom severity of the patient’s pre-post ratings decreased substantially, and her affect integration increased, indicating a much better capability to contain, tolerate, and express her feelings and thoughts when facing difficulties underlining constructive repairing of ruptures and the finding of new IP oscillations in phase 3.

### 4.4 Analytical comparison of IP and rupture repair processes within sessions

By employing a multimodal naturalistic data-driven interaction analysis to look at how behavior in the therapeutic process was continually unfolding during moments of increased HR REC, potential underlying processes of navigating the alliance were identified. The microanalysis highlighted the importance of context sensitivity in navigating emotional distress (Muntigl, 2020). Thus, a primary finding of this study was, that negotiating ruptures relied heavily on *how* they were addressed and managed during interaction. Ruptures can be described as temporary losses of we-mode between the patient and the therapist, understood as a temporary loss of their joint intentionality, while repair can be described as the cocreation of joint attention. In this study the restoring to collaborate and work together with different perspectives to create new meaning was found to take place and rely on different levels of interaction (Fonagy et al., 2023). In the investigated segments the therapist’s contribution to constructive resolution was characterized by a pendulating dance between addressing or exploring ruptures and emotion regulation strategies represented through nonverbal markers of nods, smiles, and initiated laughter (Pomeroy, 2013), and verbal strategies including the therapist’s use of confirmation, epistemic markers (Muntigl, 2020), rephrasing formulation (Antaki, 2008), relocating formulations (Antaki, 2008), highlighting formulation, active retreat (Muntigl, 2020), and emotional inference (Peräkylä, 2019). These regulatory moves while addressing and exploring ruptures were found to keep tension balanced making the interacting partners able to move forward in the meaning-making process. In this process of negotiating the alliance, increased HR REC was found to underline important processes including security, empathy, relatedness, sense-making, consolidation, affiliation, exploration, validation, and emotion regulation. While inconsistencies are present in the field (Reich et al., 2014), the majority of published studies show similar underlying functionalities of corresponding HR patterns for the patient and therapist during rupture repair episodes (Høgenhaug et al., 2024; Mylona and Avdi, 2021).

An interesting area to investigate in future work is the gain of increased IP in the clinical process. Although most studies, including ours with the investigation of sessions with maximum IP, have favored the perspective of a correlation between increased synchronization, alliance quality, and better outcome, the optimal level of synchronization between patient and therapist has still not been clarified and studies have started to show that high synchronization may not always be beneficial (Kleinbub et al., 2020). Building upon the theory of mentalization, too much or too little arousal has been found to affect the quality of mentalization negatively, resulting in breakdowns or “stuckness” in therapeutic processes (Luyten and Fonagy, 2015). Transferred to the concept of synchronization, it is likely that too much or too little attunement during rupture and repair episodes might also at times undermine therapeutic growth. Thus, upholding a homeostatic balance during moments of distress might be crucial to secure the developmental process of progress (Reed et al., 2015).

### 4.5 Strengths and limitations

The strength of this study is its multidisciplinary approach that allowed for nuanced and detailed descriptions of the correspondence between rupture repair segments and physiological responses both across and within sessions. The overall treatment process analysis identified important patterns of change across time, and the microanalysis revealed how the verbal and observable therapeutic interaction and physiological responses might be bridged and have the potential to disclose important interactional dynamics in moments of distress. Nevertheless, our results must be read in the light of their limited generalizability, as they stemmed from analyses of a single patient-therapist dyad and emerged from a positive therapeutic outcome context. Future researchers should include a larger data pool to further understand the diversity of the interplay between IP and rupture repair sequences.

Another reflection is, how the inclusion of sessions with high REC detected with Cross-RQA might have influenced the results of the multimodal interaction analysis, since other inclusion criteria would possibly have led to the identification of other dynamics. This could be a relevant topic for future work. One important thing to note about RQA is that this kind of statistical data analysis requires the theorization of a quantitative model rather than a top-down approach to interpret the findings from the data; a thorough understanding of both dynamical systems and analysis techniques is necessary to reach reliable results (Orlando et al., 2021; Orsucci, 2021).

## 5 Future research and conclusion

As described in the introduction, the field is still inconsistent according to the developmental process of ruptures in BPD treatment (Gersh et al., 2018; Schenk et al., 2019). Hence, this study may add to the perspective that different rupture processes occur in different treatments depending on the pathology or treatment method and at different times in the therapeutic process across time (Baillargeon et al., 2012). Our findings emphasize the importance of attending to moment-to-moment sequences in therapeutic interactions, as challenging events contain potential insights in relation to scaffolding a process of openness to learning (epistemic trust), affect integration, emotion regulation, and sense-making.

On a theoretical level, the results reveal how a correspondence between rupture repair events and physiological arousal can be bridged and how data-driven methodological analysis can shed new light on important interactional dynamics in clinical practice. Verbal and nonverbal levels of interactions cannot be fully understood without taking the other into account, which underscores the value of integrating physiological data in research. The results of this study indicate that IP underlines processes of different types of collaboration and relatedness which in future work should be elaborated to increase our understanding of how to facilitate processes of turning off hypervigilance and take in new knowledge during challenging moments of interaction. As shown in this study, examinations of how IP might reflect rupture repair segments have the potential to provide new perspectives on effective/ineffective factors during therapeutic interactions and could help identify why the therapist is or is not particularly attentive to exploration, negotiation, and reparation.

In future work we recommend that research should not only replicate the findings of this single-case study on bigger samples of patient-therapist dyads, taking into account different diagnoses and different therapeutic approaches, but should also examine the impact of alliance ruptures onto the further course of the treatment. The question is, if more or less successful rupture resolutions will have an impact on the therapeutic content and alliance quality of the following sessions and/or on the everyday experiences in between the sessions (Schöller et al., 2018). The impact of ruptures may also be classified like the ruptures (e.g., confrontation or withdrawal), which could allow to understand ruptures as significant events during the process (Elliott, 2010).

Additionally, it would be helpful to realize video-based micro-interviews with patient and therapists to understand the different perspectives onto the same interactional sequence. As we know, the perspectives onto and meanings of significant events may be different between therapist and patient (Elliott, 2010). A more practice-related approach for future research would be to elaborate on online-coding of verbal and nonverbal behavior from video tapes based on methods of Artificial Intelligence (AI). It would be possible not only to distinguish confrontation or withdrawal ruptures, but specific idiosyncratic patterns of confidential or doubtful/unsecure/disturbed alliance. Methods of AI-based analysis are available (Kolenik et al., 2024), and therapists could be trained in how they should react on such type of in-time feedback.

Finally, with reference to an existing theory of change, a *decision support tool* can be provided which shows the potential impact of interventions – ruptures may be seen as kind of unintended or unconscious interventions – in the phase space of a theory of change. The dimensions of the theory are “experienced success or progress,” “intensity of emotions (positive or negative),” “problem severity,” “motivation for change,” and “insight/new perspectives” (Schiepek et al., 2017; Schöller et al., 2018). In a technical sense, ruptures may be sudden dislocations of a trajectory in this 5-dimensional space, which have precursors and consequences, e.g., changed dynamic patterns. Current methods of monitoring psychotherapy (e.g., the Synergetic Navigation System) are able to measure und visualize the trajectory of change based on daily self-ratings. The time series of the items and of the dimensions (factors) can be visualized whenever the therapist wants and can see the effects of a crisis within a session in the personal experience of the patient out of the session. In routine practice, patient and therapist together reflect on the ongoing process and can also refer to the crises or interactive problems during the sessions.

Although much work is still needed to integrate such knowledge into theoretical models and clinical practices, it is found to have great potential in supervision and BPD treatment applications. First, IP methods and video coding of verbal and nonverbal behavior validate the occurrence of interpersonal crises and crisis-repair sequences during sessions. This is different from the subjective impression of crises as experienced by the therapist only. A multi-methods approach may help to validate the crisis-repair construct with all clinical consequences for tailoring the therapeutic process. Especially when using high-frequency ambulatory assessment potential consequences of alliance crises onto the cognitions, emotions, and personal experiences of the patient are represented by diagrams (e.g., time series) and transcript entries which are a useful base for common reflections. Thus, there is a potential of integrating technologies that provide direct, live feedback during therapy sessions for use *in situ* to examine co-regulation processes between the interacting partners, or it might be applied in supervision to provide more accurate feedback on alliance processes, which could strengthen the examination and training of interventions guiding alliance formation (Eubanks-Carter et al., 2015). Process feedback also grasps the quality of the ongoing working alliance. The increased frequency of alliance crises in BPD can be seen as an opportunity of interpersonal learning, particularly if the quality of the therapeutic alliance is continuously assessed. Continuous feedback interviewing establishes a routine in alliance reflection which not only takes place in emotionally charged crises, but regularly.

## Data Availability

The datasets presented in this article are not readily available because it contains participants identifiable data about patients and therapists. Requests to access the datasets should be directed to s.v.steffensen@sdu.dk.
