# Supplementary material for: The complexity of interpersonal physiology during rupture and repair episodes in the treatment of borderline personality disorder: a proof-of-concept multimethod single case study of verbal and non-verbal interactional dynamics
Source: Front Psychol. 2024 Sep 24;15:1408183. doi: 10.3389/fpsyg.2024.1408183 (PMC11462340; doi:10.3389/fpsyg.2024.1408183)
Supplement: Supplementary file 1 [file Data_Sheet_1.DOCX]

Supplementary Material

The Complexity of Interpersonal Physiology During Rupture Repair Episodes in the Treatment of Borderline Personality Disorder: A Multi Method Single Case Study

S. S. Høgenhaug^*^, S. V. Steffensen, F. Orsucci, G. Zimatore, G. Schiepek, M. T. Kongerslev, A. Bateman, and G. Kjaersdam Telléus

*** Correspondence:** Stine Steen Høgenhaug, Clinic North, Psychiatric Hospital, Hjorringvej 180, 9700 Bronderslev, Denmark, Department of Clinical Medicine, Faculty of Medicine, Aalborg University, Aalborg, Denmark. Email: [s.hoegenhaug@rn.dk](mailto:s.hoegenhaug@rn.dk). Telephone: +4560138709.

# Overview of data availability. *All and SCL-92.

| Session N | 3RS | HR Couple Therapist/Sophie | Outcome* |
| --- | --- | --- | --- |
| 1 |  |  |  |
| 2 |  |  |  |
| 3 | X | S03TP |  |
| 4 | X | S04TP |  |
| 5 | X | S05TP | 11/03/19 |
| 6 | X | S06TP |  |
| 7 | X | S07TP |  |
| 8 | X | S08TP |  |
| 9 |  |  |  |
| 10 |  |  |  |
| 11 | X | S11TP |  |
| 12 | X | S12TP |  |
| 13 | X | S13TP |  |
| 14 | X |  | 20/06/19 |
| 15 | X | S15TP |  |
| 16 | X |  |  |
| 17 | X | S17TP |  |
| 18 | X |  | 28/08/19 |
| 19 | X | S19TP |  |
| 20 | X |  |  |
| 21 | X |  |  |
| 22 |  |  |  |
| 23 |  |  |  |
| 24 | X | S24TP |  |
| 25 | X | S25TP |  |
| 26 |  |  |  |
| 27 | X |  |  |
| 28 | X | S28TP |  |
| 29 | X | S29TP |  |
| 30 | X | S30TP |  |
| 31 | X | S31TP |  |
| 32 | X | S32TP |  |
| 33 | X | S33TP |  |
| 34 | X | S34TP | 08/02/20 |
| Total 34 | 28 | 21 | 5 |
| Follow up* |  |  | 02/08/20 |

# Recurrence Quantification Procedure

To briefly introduce the measurement procedure in RQA, a time series is embedded into a higher-dimensional phase space (here, the embedding dimension was fixed at 15). This involves creating a trajectory by plotting the time-delayed versions of the time series against each other. Successively, a recurrence plot is created by determining when states in the trajectory revisit or recur. A binary matrix is formed where a “1” (a black point drawn on the recurrence plot; in this study, radius = 25) indicates recurrence, and a “0” indicates non-recurrence. Two measures were calculated based on RP, including: recurrence, or the proportion of recurrent points in the phase space, and determinism, the proportion of recurrent points forming diagonal lines of a certain length (here, a line = 40). The Python Software packages were used. The analysis of the chaotic and stochastic dynamics of the physiological signals (i.e., HR) using RQA has proved to be an accurate approach (Zbilut, Thomasson, & Webber, 2002).

# HR REC for the patient over the course of treatment

**Figure 2.** *Percentage of recurrence points (% REC) derived from the analysis of Sophie’s HR time series recorded during the SCL-92 sessions*


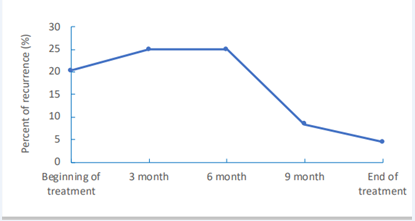


*Note.* The session dates considered are the same as in Figure 1 in the paper.

Per the SCL-92’s GSI (Figure 1), results revealed that after 6 months, the HR had a smaller percentage of recurrence points during sessions (the % REC diminished; Figure 2). Specifically, the *determinism*—defined as a measure that captures the rate of recurrence points forming diagonal lines —decreased after Session 11. This is comparable to research indicating that health of the heart (detectable by HR) is inversely correlated with determinism (Zbilut, Thomasson, & Webber, 2002). The increased determinism at the beginning of treatment could represent more rigid and less flexible physiological responses where the patient struggled to maintain stability in the face of difficulties. The decreased determinism after 6 months might suggest an ability to adapt more flexibly to changing circumstances and stress, comparable to the results from the AII.

# Danish transcriptions of included segments

## Session 3

**Minute 10: mirroring sadness.**

P: så (0.6) så det var det var et rigtig hårdt slag for os alle sammen som (0.4) jeg stadigvæk ikke helt har fundet ud af hvordan jeg skal håndtere

T: mm (0.4) nej

P: nej (…)

T: men jeg kan se, at det gør dig ked af det

P: ja hæhhh ja det gør det .hhh

T: ja

P: øhmm fordi hun er primus motor på så mange ting

T: mm

P: øh (1.1) og og ud af mine bedsteforældre er det hende jeg har det tætteste forhold til

## Session 5

**Minute 8: brave**

T: men hold da op, hvor har du været modig

P: ja hæ:hæ:

T: synes jeg

P: det [øh]

T: [faktisk]

P: det var en stor dag

T: altså, du gjorde jo (0.6) for lige at highlighte det lidt

P: ja

T: du gjorde noget andet end at barrikadere dig

P: ja

T: og gemme dig bag din forsvarsmur

P: ja

T: ikke også

P: ja

T: du gjorde noget andet end du (0.3) plejer

P: ja

T: og end du ville have gjort

P: ja (0.6) de:t øh(2.5) ja (0.5) altså, det, det at have et eller andet sted styrken til at, tage sted selvom (0.6) risikoen forelå at

T: ja

P: at jeg bare ville blive afvist. Det øh (1.4) det var stort

## Session 33

**Minute 1-2: addressing a rupture**

T: ja (1.8) men der er noget, der måske er uafsluttet (0.2) fra gruppen eller hvad

P: .hhh ja hæ:hæhæ [ja]

T: [jeg] skal jo ikke definere hvad vi skal snakke om i dag

P: [nej]

T: [Det] kan være det ikke fylder for dig

P: øh det gør [det].

T: [men] jeg tænkte, at det gør det nok

P: ja

T: [ja]

P: [Jeg] øh hhh øh jeg jeg gik derfra med en masse skam over at have mistet kontrollen jeg brød mig virkelig ikke om

T: [okay]

P: [øhm] jeg tror, jeg fik ident, altså jeg fik både identificeret, og det gjorde virkelig virkelig ondt at (0.7) den måde det jeg havde sagt blev vendt på

T: ja

P: og brugt

T: ja

P: og især at (0.1) du sagde det gjorde rigtig rigtig ondt

T: ja

P: øh (0.7) og så det at jeg føler, at jeg har mistet kontrollen foran en som jeg faktisk ikke stoler på (0.7) lige nu altså foran Tina

T: nå foran Tina

P: jahhh øh det bryder jeg mig ikke om

T: nej (0.6) på den måde

P: ja

T: ja
