# Supplementary material for: The complexity of interpersonal physiology during rupture and repair episodes in the treatment of borderline personality disorder: a proof-of-concept multimethod single case study of verbal and non-verbal interactional dynamics
Source: Front Psychol. 2024 Sep 24;15:1408183. doi: 10.3389/fpsyg.2024.1408183 (PMC11462340; doi:10.3389/fpsyg.2024.1408183)
Supplement: Supplementary file 2 [file Table_1.DOCX]

| **Session** | **Segment** | **Recurrence measure in HR** | **Verbal and nonverbal cues** | **IP underlining the following alliance processes** | **Rupture/repair** |
| --- | --- | --- | --- | --- | --- |
| 3* | Minute 10 | Therapist (T) increased REC showing potential correspondence to Sophie’s (S) increased REC in minute 20 | Therapists use of emotional inference | Empathy  Shared emotional experience  Security | Withdrawal |
| 3 | Minute 20 | S’s increased REC showing potential correspondence to T’s increased REC in minute 10 | Overlapping talk  Therapists use of continuers | Sense-making | None |
| 3 | Minute 23 | T’s increased REC showing potential correspondence to S’s increased REC in minute 33 | Therapists use of emotional inference | Empathy | None |
| 3 | Minute 33 | S’s increased REC showing potential correspondence to T’s increased REC in minute 23 | Therapists use of emotional inference and continuers | Empathy  Shared emotional experience  Security | Withdrawal |
| 5* | Minute 8 | Simultaneous increased REC responses in T and S | Therapists initiated shared laughter and use of highlighting formulations | Validation  Affiliation  Consolidation | None |
| 5 | Minute 25 | Simultaneous increased REC responses in T and S | Therapists use of deep nods and  continuers | Empathy | None |
| 13 | Minute 3.5 | Lagged independent increased REC responses initially for T, followed by S | Therapists use of prompting | Elaboration  Emotional dysregulation | Confrontation, withdrawal, repair |
| 29 | Minute 2.5 | T’s increased REC showing potential correspondence to S’s increased REC in minute 20 | Therapists use of continuers and summarizing | Empathy | None |
| 29 | Minute 13 | T’s increased REC showing potential correspondence to S’s increased REC in minute 27 | Therapists use of continuers | Empathy | None |
| 29 | Minute 20 | S’s increased REC showing potential correspondence to T’s increased REC in minute 2.5 | Therapists use of rephrasing formulations | Empathy  Sense-making  Validation | Confrontation, withdrawal, repair |
| 29 | Minute 27 | S’s increased REC showing potential correspondence to T’s increased REC in minute 13 | Therapists use of relocating formulations | Sense-making | Confrontation, withdrawal, repair |
| 33* | Minute 0.5 | Lagged increased REC with precisely 1 minute between peaks in S’s and T’s HR response | Therapists use of retreat, deep nods and  continuers | Empathy  Validation  Emotion regulation  Affiliation  Security | Confrontation, withdrawal, repair |
| 33 | Minute 19 | Lagged increased REC with precisely 1 minute between peaks in S’s and T’s HR response | Patient’s initiated laughter | Emotion regulation | Confrontation, withdrawal, repair |
| 33 | Minute 24 | Lagged increased REC with precisely 1 minute between peaks in S’s and T’s HR response | Therapist initiated laughter | Shared emotional experience  Sense-making  Validation  Security | Confrontation, withdrawal, repair |
